# Direct needle puncture technique using a radial miniature probe for complete anastomotic obstruction after hepaticojejunostomy

**DOI:** 10.1055/a-2550-3875

**Published:** 2025-03-12

**Authors:** Tomohiro Ishii, Kazuya Sugimori, Michio Ueda, Arisa Omata, Shoichiro Yonei, Takashi Kurosawa, Shin Maeda

**Affiliations:** 1 89460 Gastroenterology, Saiseikai Yokohamashi Nanbu Hospital, Yokohama, Japan; 2 89460 Surgery, Saiseikai Yokohamashi Nanbu Hospital, Yokohama, Japan; 3 Gastroenterology, Yokohama City University Graduate School of Medicine, Yokohama, Japan

Anastomotic stenosis of the hepaticojejunostomy is a major complication following pancreaticoduodenectomy 1 . In cases of complete anastomotic obstruction, drainage has been attempted using a cholangioscope via the percutaneous transhepatic biliary drainage route or a forward-viewing echoendoscope 2 3 . However, reports of direct puncture of the anastomotic site remain scarce 4 . Here, we present a case of complete hepaticojejunostomy obstruction successfully managed with direct needle puncture drainage using a radial miniature probe (UM-2R; Olympus, Tokyo, Japan).

A 72-year-old woman was under postoperative surveillance following surgery for pancreatic tail cancer when an iatrogenic recurrence of pancreatic head cancer was detected. She subsequently underwent residual pancreatectomy. Postoperatively, she developed obstructive jaundice ( Fig. 1 and Fig. 2 ) and was referred to our department. Single-balloon enteroscopy (SIF-H290S; Olympus, Tokyo, Japan) was performed to evaluate the hepaticojejunostomy anastomosis; however, complete occlusion precluded biliary drainage. The endoscope was then switched to a therapeutic video gastroscope (GIF-H290T; Olympus, Tokyo, Japan) to access the anastomotic site. Water was slowly injected through the auxiliary water channel, and the water-filled area surrounding the hepaticojejunostomy was visualized using the UM-2R. The right hepatic artery and portal vein were clearly identified, confirming a safe trajectory for puncturing the anastomosis and accessing the bile duct.

**Fig. 1:**
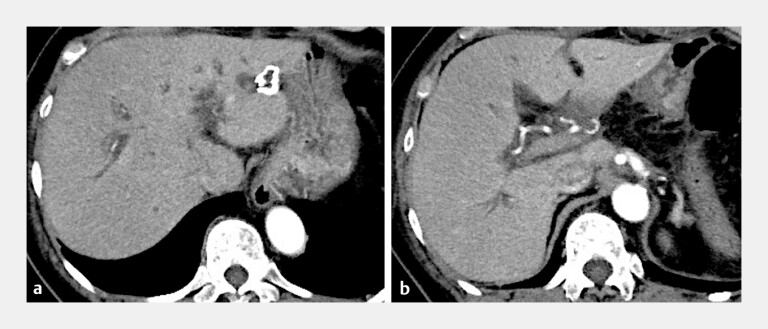
Arterial phase of contrast-enhanced computed tomography. **a** Dilatation of the intrahepatic duct upstream of the hepaticojejunostomy anastomosis. **b** No tumor recurrence was noted near the hepaticojejunostomy anastomosis. The right hepatic artery and portal vein are adjacent to the anastomosis.

**Fig. 2:**
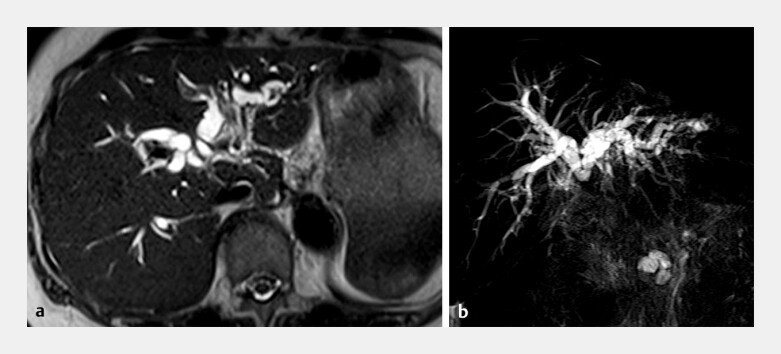
**a–b** T2-weighted image ( **a** ) and magnetic resonance cholangiopancreatography ( **b** ) showing intrahepatic duct dilation.

A 19-G needle was used to directly puncture the hepaticojejunostomy anastomosis, allowing successful cholangiography. Following guidewire placement, the anastomotic stenosis was dilated with a biliary balloon catheter, and 7-Fr biliary stents were placed in the right and left hepatic ducts, achieving effective drainage ( Video 1 ).

A 72-year-old woman with anastomotic stenosis of hepaticojejunostomy underwent dilatation with a biliary balloon catheter. The right hepatic artery and portal vein were identified, and the direction of puncture from the anastomosis to the bile duct was confirmed to be safe Subsequently, 7-Fr biliary stents were placed in the right and left hepatic ducts, achieving effective drainage.Video 1

Direct needle puncture of the hepaticojejunostomy anastomosis carries potential risks, including small intestinal perforation, bile leakage, and vascular injury leading to hemorrhage. However, the use of the UM-2R enabled safe bile duct access and drainage while mitigating complications, thus broadening the scope of endoscopic management for anastomotic stenosis.

Endoscopy_UCTN_Code_TTT_1AS_2AH

## Notes

Endoscopy E-Videos https://eref.thieme.de/e-videos
*E-Videos* is an open access online section of the journal *Endoscopy* , reporting on interesting cases and new techniques in gastroenterological endoscopy. All papers include a high-quality video and are published with a Creative Commons CC-BY license. Endoscopy E-Videos qualify for HINARI discounts and waivers and eligibility is automatically checked during the submission process. We grant 100% waivers to articles whose corresponding authors are based in Group A countries and 50% waivers to those who are based in Group B countries as classified by Research4Life (see: https://www.research4life.org/access/eligibility/ ). This section has its own submission website at https://mc.manuscriptcentral.com/e-videos .
